# Numerical Modeling of the Photothermal Processing for Bubble Forming around Nanowire in a Liquid

**DOI:** 10.1155/2014/794630

**Published:** 2014-03-24

**Authors:** Anis Chaari, Laurence Giraud-Moreau, Thomas Grosges, Dominique Barchiesi

**Affiliations:** Group for Automatic Mesh Generation and Advanced Methods, Gamma3 (UTT-INRIA), University of Technology of Troyes, 12 rue Marie Curie, CS 42060, 10004 Troyes Cedex, France

## Abstract

An accurate computation of the temperature is an important factor in determining the shape of a bubble around a nanowire immersed in a liquid. The study of the physical phenomenon consists in solving a photothermic coupled problem between light and nanowire. The numerical multiphysic model is used to study the variations of the temperature and the shape of the created bubble by illumination of the nanowire. The optimization process, including an adaptive remeshing scheme, is used to solve the problem through a finite element method. The study of the shape evolution of the bubble is made taking into account the physical and geometrical parameters of the nanowire. The relation between the sizes and shapes of the bubble and nanowire is deduced.

## 1. Introduction

In the last years, many researchers are interested in the use of nanomaterials. In the chemical industry and in the manufactures of nanotubes and nanowires, the usually used materials are TiO_2_ and ZnO [1–3]. Such a use of these nanomaterials (natural or artificial) increases and these are dispersed in air or in water [4]. Their impact on the environment and health must be evaluated (e.g., toxicity analysis) [5]. Therefore, the detection of the presence of such nanomaterials in the environment becomes crucial. Two modes of detections of such a nanowire/nanotube can be achieved. The first one consists in a direct detection of the nanowire by optical microscopy through the measurement of the scattering of light emitted by the nanomaterial. Due to a weak signal/noise ratio, such a detection mode can be difficult. The second mode is an indirect method and consists in studying the bubble created by the photothermal response of the nanowire immersed in a liquid and illuminated by an electromagnetic wave. In such an approach, the nanowire absorbs the electromagnetic radiation (energy) for a range of wavelengths and heats and, for temperature exceeding the threshold of vaporization of the liquid, induces the creation of a nanobubble [6, 7]. The created bubble grows before being detected. The analysis of the shape and size of the bubble should permit studying the morphology of the nanowire. The studied problem consists in solving a photothermic coupled system (light, nanowire, and heat) taking into account the physical parameters of the system (i.e., permittivity of materials, material conductivity, laser wavelength, and laser power).

In that context, a numerical multiphysic model, allowing studying the behavior of the nanowires illuminated by an incident laser field, is presented. The formation of the bubble, associated with a nanowire of TiO_2_ immersed in water and illuminated by a laser pulse, is studied. An optimization process, including adaptive remeshing scheme, is used to detect the variations of the temperature, the bubble shape evolution, ensuring the convergence of the solution to the physical solution [8, 9]. The paper is organized as follows.  Section 2 describes the equations of the model and the numerical resolution method. The adaptive remeshing process and the optimization steps are presented in Section 3. In Section 4, the results of numerical simulations are presented before concluding.

## 2. Model and Numerical Methods

The section is devoted to presenting the equation systems modeling the photothermic process and the numerical method used to solve the system.

### 2.1. Electromagnetic Problem

In electromagnetic system, the partial differential equations are derived from Maxwell's equations. The problem can be reduced to Helmholtz equation for the harmonic electric **E** and magnetic **H** fields (i.e., in the form exp⁡(*jωt*), where *ω* is the angular frequency of the harmonic wave) [10]. In the 2D case of an infinity elliptical cylinder along the *z*-axis, the unknown field is the magnetic component and, for a polarized illumination in the transverse magnetic mode TM, the magnetic field can be written as **H**(*x*, *y*) = (0,0, *H*_*z*_(*x*, *y*)). Therefore, the electromagnetic problem is reduced to a scalar problem and the computation of **H**(*x*, *y*), in a domain *Ω*, allows deducing the electric field **E**(*x*, *y*) by using the Maxwell-Ampere equation [10]:

(1) $$\begin{array}{c} E\left(x,y\right)=\frac{-j}{\omega \epsilon_{r}\epsilon_{0}}\left[\nabla \times H\left(x,y\right)\right],\;\forall \left(x,y\right)\in \Omega , \end{array}$$

where (∇×·) is the rotational operator, *ω* the angular frequency, *ϵ*_0_ the permittivity of vacuum, and *ϵ*_*r*_ the relative complex permittivity of the considered materials which are functions of the spatial coordinates (*x*, *y*). The *H*_*z*_ component of the magnetic field satisfies the scalar equation

(2) $$\begin{array}{c} \left[\nabla .\left(\frac{1}{\epsilon_{r}}\nabla \right)+k_{0}^{2}\right]H_{z}\left(x,y\right)=0,\;\forall \left(x,y\right)\in \Omega , \end{array}$$

where *k*_0_ = *ω*/*c* is the wave number of the monochromatic incoming wave and *c* the speed of light in vacuum. To compute the solution *H*_*z*_(*x*, *y*) of the electromagnetic problem, a set of conditions on the boundary Γ of the computational domain *Ω* must be imposed. The natural boundary condition at the interface between materials is the continuity of the normal component of the electromagnetic excitation:

(3) 1ϵr∂Hz(x,y)∂n=−jk0[Hz(x,y)−(ny−1)Hi(x,y)],∀(x,y)∈Γ,

where ∂/∂*n* is the normal derivative operator, *n*_*y*_ is the normal vector component along the *y*-axis, and *H*_*i*_ = *H*_0_exp⁡(*jk*_0_ *y*) is the incident illumination field along the *y*-axis with *H*_0_ = 1/(*cμ*_0_) and *μ*_0_ being the permeability of vacuum. Such a boundary condition is used in problems of wave propagation [11–13].

### 2.2. Thermic Problem

Under illumination by an electromagnetic wave, the nanowire absorbs energy. That energy produces a heat source given by

(4) $$\begin{array}{c} Q\left(x,y\right)=\frac{\omega }{2}\epsilon_{0}Im\left(\epsilon_{r}\right){\left|E\left(x,y\right)\right|}^{2},\;\forall \left(x,y\right)\in \Omega . \end{array}$$

The resolution of the thermal problem requires solving the heat equation which is a partial differential parabolic equation describing the evolution of the temperature *T* with a heat source *Q*. That equation is written as follows:

(5) $$\begin{array}{c} \left[\nabla \cdot \left(k\left(x,y\right)\nabla \right)\right]T\left(x,y\right)=Q\left(x,y\right),\;\forall \left(x,y\right)\in \Omega , \end{array}$$

with a Dirichlet boundary condition *T* = *T*_0_ and *k*(*x*, *y*) is the thermal conductivity of the materials. The variation of the temperature depends on both imaginary part of the permittivity *ϵ*_*r*_(*x*, *y*) and the intensity of the electric field |**E**(*x*, *y*)|^2^.

The resolution of the coupled electromagnetic and heat problems allows extracting the spatial distribution of the temperature in the computational domain. From the map of temperature and for a fixed threshold of vaporization *α*, the identification of the shape and size of the bubble around the nanowire can be achieved. Such information on shape and size of the bubble would be used to construct a relation between the geometric characteristics of the bubble and the nanowire.

### 2.3. The Finite Element Method

The objective is to solve (2) and (5) for the coupled system in a domain whose geometry can be complex. The Finite Element Method (FEM) was applied since the 1940s in mechanics, thermodynamics, electromagnetics, and electrical engineering [14, 15]. The method is used to solve partial differential equation systems with boundary conditions in open or close domains. The resolution of problem necessitates a discrete domain, generally named mesh of the domain [13]. The solutions of the problem are computed on the nodes of the mesh. In order to both control the error on the solution and to decrease the number of nodes, an improved method, including an iterative remeshing process, is developed and used. Such an improved FEM allows describing the complex structures with arbitrary shapes. Moreover, the stability of the FEM is also improved by using a weak formulation (or variational formulation) of (2) and (5). Therefore, the electromagnetic and thermic fields satisfy

(6) $$\begin{array}{c} \int_{\Omega }\left[\nabla \cdot \left(\frac{1}{\epsilon_{r}}\nabla H_{z}\left(x,y\right)\right)+\frac{\omega^{2}}{c^{2}}H_{z}\left(x,y\right)\right]\cdot \nu d\Omega =0,\\ \int_{\Omega }\left[\nabla \cdot \left(k\left(x,y\right)\nabla \right)T\left(x,y\right)-Q\left(x,y\right)\right]\cdot \tilde{\nu }d\Omega =0, \end{array}$$

where *ν* and $\tilde{\nu }$ are test functions defined on *L* ^2^(*Ω*) (the linear space of the scalar functions *ν* and $\tilde{\nu }$, being 2-integrable on *Ω*). The basis of polynomial functions provides an approximation of the solutions *H*_*z*_ and *T* in each node [16]. The field *H*_*z*_ (resp., *T*) is a linear combination of such basic polynomial functions *ν* (resp., $\tilde{\nu }$) and the problem consists in solving a linear system [15, 17]. The solution verifies exactly the partial differential equations on each node for the given boundary conditions. Ritz's formulation of the variational problem is used to satisfy the continuity of the tangential components of the electromagnetic field [13].

## 3. Optimization Process and Adaptive Remeshing

Partial differential equations (electromagnetic and thermic) are formulated and solved on the mesh of the computational domain through the FEM. But the accuracy of the computed solution depends on the quality of the mesh [9, 18, 19]. A remeshing process and adaptive loops have been developed in order to improve the quality of the solutions by adapting the size of the mesh elements to the physical solution [9, 20]. The mesh adaption is required to converge to a stable solution, in particular where strong variation of the electromagnetic or temperature fields occurred. For each step of the adaption process, the approximate solution of the Helmholtz equation, the electric field **E**, the heat source *Q*, and the temperature *T* are computed [20]. The interpolation error, based on an estimation of the discrete Hessian of the solution, is used to limit the maximum deviation between the exact solution and the solution associated with the mesh [21, 22]. The a posteriori error estimator, based on the interpolation error, allows defining a physical size map *C*_*p*_(*Ω*) such as

(7) $$\begin{array}{c} C_{p}\left(\Omega \right)=\left\{h_{p}\left(x,y\right)\right\},\;\forall \left(x,y\right)\in \Omega , \end{array}$$

where *h*_*p*_(*x*, *y*) is the physical size defined at each node and is proportional to the inverse of the deviation of the Hessian. For a given maximum tolerance on the physical error *γ*, the size *h*_*p*_(*x*, *y*) is given by

(8) $$\begin{array}{c} h_{\min}\leq h_{p}\left(x,y\right)=\frac{\gamma }{\eta \left(x,y\right)}\leq h_{\max}, \end{array}$$

where *h*_min⁡_ and *h*_max⁡_ are the minimum and maximum sizes of the elements and *η*(*x*, *y*) is an estimation of the maximum deviation obtained from the Hessian of the solution. The physical size map *C*_*p*_(*Ω*) is used to govern the adaptive remeshing of the domain with the BL2D-V2 software (adaptive remeshing generating isotropic or anisotropic meshes) [23]. The domain is then entirely remeshed and a new mesh *M*_*p*_(*Ω*) is obtained. The resolution of the multiphysics problem is based on the computation of two physical size maps: the first one *C*_*Q*_(*Ω*) related to the heat source *Q* and the second one *C*_*T*_(*Ω*) related to the temperature *T*. The adaptive computational scheme consists in iterative and adaptive loops:

*A*_1_ initial mesh *M*_*i*=0_(*Ω*) generated with triangular elements of the computational domain *Ω*,
*A*_2_ computation of the field (*H*_*z*_)_*i*_ (solution of (2)) on *M*_*i*_(*Ω*),
*A*_3_ computation of the solutions **E**_*i*_ and *Q*_*i*_ on *M*_*i*_(*Ω*),
*A*_4_ physical error estimation: computation of the interpolation error of the physical solution *Q*_*i*_; definition of a physical size map *C*_*Q*_*i*__(*Ω*) connected to the field *Q*_*i*_ enabling relating the error to a given threshold *δ*,
*A*_5_ remeshing of the domain conforming to the size map *C*_*Q*_*i*__(*Ω*),
*A*_6_ if the threshold *δ* is not satisfied loop to step *A*_2_, with *i* = *i* + 1, in order to obtain a new mesh *M*_*i*_(*Ω*),
 else *M*_*Q*_(*Ω*) = *M*_*i*_(*Ω*), and *M*_*i*=0_(*Ω*) = *M*_*Q*_(*Ω*),
*B*_1_ computation of the solutions *T*_*i*_ on *M*_*i*_(*Ω*),
*B*_2_ physical error estimation: computation of the interpolation error of the physical solution *T*_*i*_; definition of a physical size map *C*_*T*_*i*__(*Ω*) connected to the field *T*_*i*_ enabling relating the error to a given threshold *δ*,
*B*_3_ remeshing of the domain conforming to the size map *C*_*T*_*i*__(*Ω*),
*B*_4_ if the threshold *δ* is not satisfied loop to step *B*_1_, with *i* = *i* + 1, in order to compute the temperature *T*_*i*_ on the new adapted mesh *M*_*i*_(*Ω*),
 else *M*_*T*_(*Ω*) = *M*_*i*_(*Ω*),
*C*_1_ detection of the new domain (water vapor) on *M*_*T*_ for a fixed threshold of vaporization in order to produce a mesh *M*_*V*_(*Ω*) and *M*_*i*=0_(*Ω*) = *M*_*V*_(*Ω*),
*C*_2_ computation of the physical solutions *T*_*i*_ on *M*_*i*_(*Ω*),
*C*_3_ physical error estimate: computation of the interpolation error of the physical solution *T*_*i*_; definition of a physical size map *C*_*V*_*i*__(*Ω*) connected to the field *T*_*i*_ enabling relating the error to a given threshold *δ*,
*C*_4_ remeshing of the domain conforming to the size map *C*_*V*_*i*__(*Ω*),
*C*_5_ if the threshold *δ* is not satisfied loop to step *C*_2_, with *i* = *i* + 1, in order to compute the temperature *T*_*i*_ on the last adapted mesh *M*_*i*_(*Ω*),
 else *M*_*F*_(*Ω*) = *M*_*i*_(*Ω*).

## 4. Numerical Results and Discussion

Here, we consider a TiO_2_ elliptical nanowire of semiaxes (*a* = 45 nm and *b* = 10 nm), with thermal conductivity *k*(TiO_2_) = 11.7 Wm^−1^ K^−1^ immersed in water (*ϵ*_*r*_(water) = 1.79 and *k*(water) = 0.6 Wm^−1^ K^−1^) at temperature *T*_0_ = 25°C(298.15 K). The nanowire is illuminated by a TM polarized laser pulse at wavelength *λ* = 1050 nm of complex permittivity *ϵ*_*r*_(TiO_2_)_1050_ = 5.4600 + *j*0.00148 with a power density per area units *P*_*S*_ = 1.75 × 10^12^ W/m^2^ [24, 25]. The materials of the system are considered isotropic and homogeneous.

**Figure 1:**
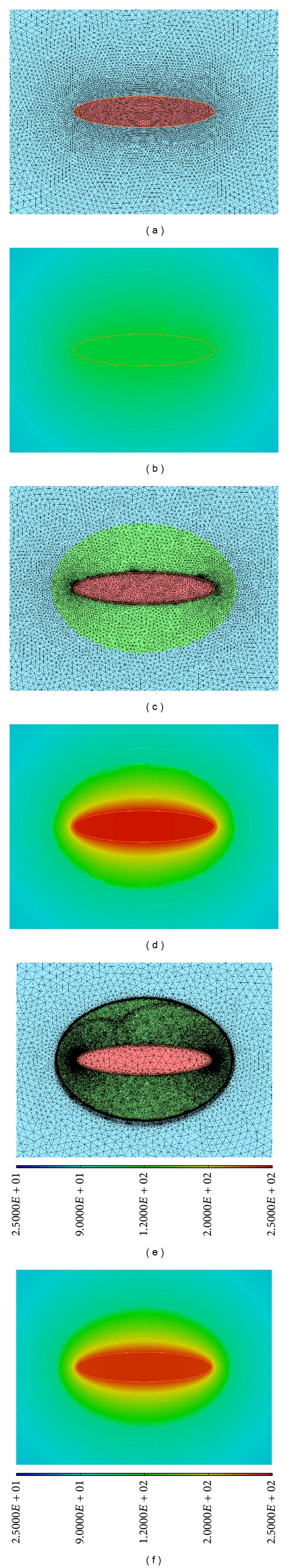
Initial mesh *M*_0_ (a) and adaptive meshes *M*_*V*_0__ (c) and *M*_*F*_ (e) and the associated temperature maps on *M*_0_ (b), *M*_*V*_0__ (d), and *M*_*F*_ (f) for nanowire illuminated at *λ* = 1050 nm.

The results of the adaptive process on mesh and on the temperature maps are illustrated in Figure 1. Figures 1(a) and 1(b) show the initial mesh *M*_0_ and the associated temperature. The adaptive process on the temperature field *T* (with *γ* = 0.0001, *h*_max⁡_ = 40 nm, *h*_min⁡_ = 0.03 nm, and *δ* = 0.1) produces the mesh *M*_*T*_ and the temperature map. The mesh is adapted on the outline of the nanowire that presents strong variations of the temperature. For a water vaporization threshold *α* = 100°C(373.15 K), the detection of the new material (water vapor) is obtained from the temperature map computed on the mesh *M*_*T*_.  Figure 1(c) presents the areas of the three materials: TiO_2_(red), vapor (green), and water (blue). The computation of the temperature on the domain that contains the water vapor requires including the physical parameters of the vapor (permittivity *ϵ*_*r*_(vap) = 1.79 and thermic conductivity *k*(vap) = 0.05 Wm^−1^ K^−1^). The spatial distribution of the temperature field *T* on the mesh *M*_*V*_0__ after detection of the bubble produced around the nanowire is shown in Figure 1(d). The final mesh *M*_*F*_ is obtained, after eight iterations, by applying the adaptive process on the field *T* (with *δ* = 0.02) taking into account the bubble. That mesh is adapted in the bubble especially on its outline where variations in the temperature occur and relaxed inside the nanowire where the temperature is almost constant (Figure 1(e)). The remeshing process takes into account the shape and size of the bubble.  Figure 1(f) shows the temperature map *T* on the mesh *M*_*F*_ after convergence to a stable solution. The level curves are smooth where a strong variation of the temperature is shown (in the vicinity of the boundary of the bubble and the nanowire). The map also shows an increase of the temperature in the nanowire due to the creation of the bubble. Such an increase is due to the diffusion of the temperature, produced by the nanowire after detection of the bubble (i.e., the water vapor has a smaller thermal conductivity than water).

**Figure 2:**
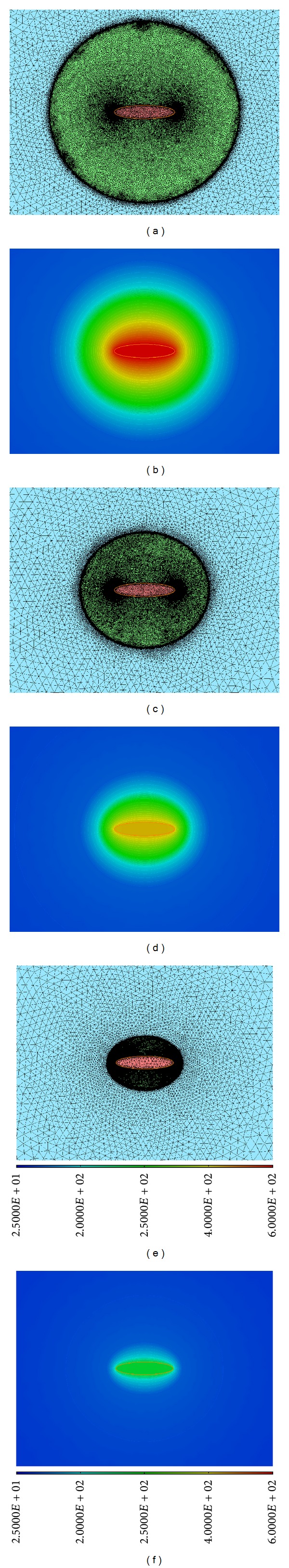
Adapted meshes *M*_*F*_ ((a), (c), and (e)) and temperature map *T* on *M*_*F*_ ((b), (d), and (f)) for wavelengths *λ* = 950, 1000, and 1050 nm, respectively.

**Figure 3:**
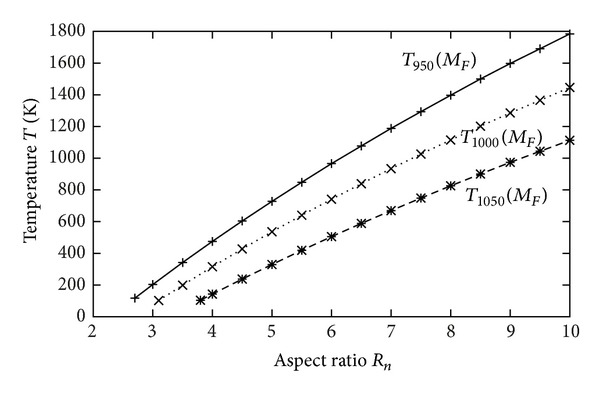
Evolution of the mean temperature as function of the nanowire aspect ratio *R*_*n*_ after formation of the bubble for the three wavelengths on *M*_*F*_.

In order to study the evolution of the shape and size of the bubble, we also consider the nanowire illuminated at three wavelengths *λ* = 950 nm, *λ* = 1000 nm, and *λ* = 1050 nm with physical parameters (*ϵ*(TiO_2_)_950_ = 5.500 + *j*0.00164, *ϵ*_*r*_(TiO_2_)_1000_ = 5.475 + *j*0.00154, and *ϵ*_*r*_(TiO_2_)_1050_ = 5.460 + *j*0.00148). Figures 2(a), 2(b), 2(c), 2(d), 2(e), and 2(f) present the mesh *M*_*F*_ after bubble detection and distribution of the temperature *T* on the meshes for the three different wavelengths *λ* = 950 nm, *λ* = 1000 nm, and *λ* = 1050 nm, respectively. These show the evolution of the meshes (shape and size of the bubble) and the temperature as function of the wavelength. For *λ* increasing, the imaginary part of the complex permittivity of the TiO_2_ decreases, leading to a decrease of the energy absorbed by the nanowire. Therefore, the temperature also decreases and the shape and size of bubble are changing. The created bubble follows the shape of the nanowire (elliptical) at the beginning and becomes circular with the increase of the temperature.  Figure 3 shows the evolution of the mean temperature *T* in the nanowire as function of the aspect ratio *R*_*n*_ = *a*/*b* for three wavelengths *λ* = 950, 1000, and 1050 nm on the mesh *M*_*F*_.

**Figure 4:**
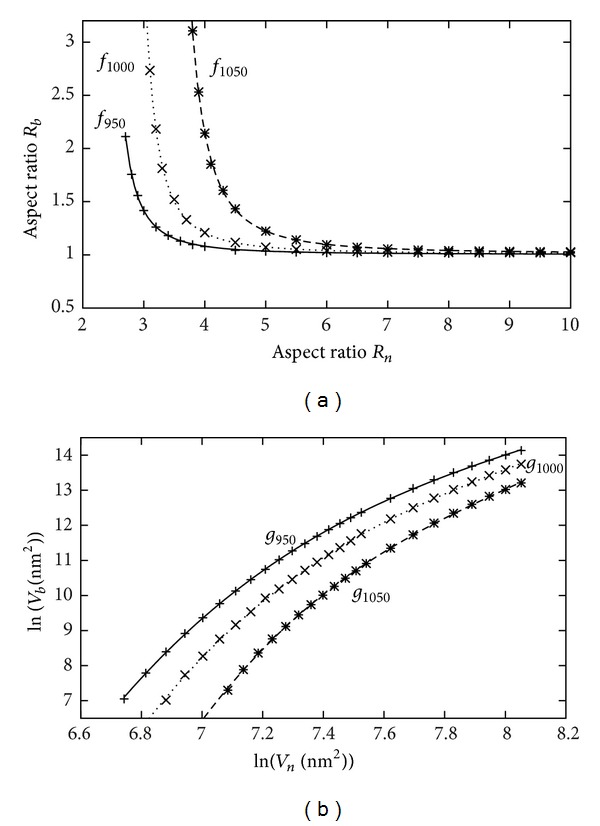
Evolution of (a) the aspect ratio of the bubble *R*_*b*_ as function of the aspect ratio of the nanowire *R*_*n*_ for the three wavelengths and (b) evolution of the volume of the bubble *V*_*b*_ as function of the volume of the nanowire *V*_*n*_.

Figure 4(a) shows the evolution of the aspect ratio of the bubble *R*_*b*_ = *A*/*B* (*A* and *B* being the semiaxes of the bubble) as function of the aspect ratio of the nanowire *R*_*n*_ for the three wavelengths. From the computed data, a function *f*, satisfying *R*_*b*_ = *f*(*R*_*n*_), can be obtained through a nonlinear least-squares fit method (LLS) by the Marquardt-Levenberg algorithm [26–29]. The method is used to find a set of the best parameters fitting the data. It is based on the sum of the squared differences or residuals (SSR) between the input data and the function evaluated at the data. The applied algorithm consists in minimizing the residual variance *σ* ^2^ = SSR/NDF with NDF being the number of degrees of freedom (number of the data points (NDP) minus number of the estimated parameters) after a finite number of iterations (FNI). Therefore, the function can be written as follows:

(9) $$\begin{array}{c} f\left(x\right)=a_{0}+\frac{a_{1}}{{\left(x-a_{2}\right)}^{2}}, \end{array}$$

with *a*_0_, *a*_1_, and *a*_2_ being set of parameters varying as function of the wavelength. The parameter *a*_0_ concerns the asymptote value which is related to the maximum ratio for a circular bubble, *a*_1_ is the inverse of the decay rate of the function *f* which is related to the speed tending to the circular shape, and *a*_2_ is the initial ratio from which the bubble begins to form.  Table 1 shows the fit parameters for each wavelength. The *f* function is continuous and strictly decreasing for *R*_*n*_ in the interval ]*a*_2_, +*∞*[; therefore the inverse function *f* ^−1^ exists. The measurement of the aspect ratio of the bubble *R*_*b*_ allows predicting the aspect ratio of the nanowire *R*_*n*_ through the relation *R*_*n*_ = *f* ^−1^(*R*_*b*_).  Figure 4(b) presents the evolution of the bubble volume (in 2D: *V*_*b*_ = *πAB*) as function of the volume of the nanowire (i.e., *V*_*n*_ = *πab*) for each wavelength. From the computed data (vapor bubble for each nanowire and for each wavelength) and by using the same method and the same algorithm, a function *g* can be obtained through a fit. That function *g* allows obtaining the relation between the volumes ln⁡(*V*_*b*_) = *g*(ln⁡(*V*_*n*_)). The *g* function is continuous and strictly increasing; therefore the inverse function *g* ^−1^ also exists. The measurement of the bubble volume *V*_*b*_ can be used to determine the volume of the nanowire *V*_*n*_ through the relation ln⁡(*V*_*n*_) = *g* ^−1^(ln⁡(*V*_*b*_)). With the two functions *f* ^−1^ and *g* ^−1^, the size and shape of the nanowire can be obtained from the information on the bubble:

(10) ln⁡(Vn)=ln⁡(πba)=ln⁡(πb2Rn)=ln⁡(πb2f−1(Rb))=g−1(ln⁡(Vb));

consequently,

(11) b=[exp⁡(g−1(ln⁡(Vb)))πf−1(Rb)]1/2,a=[f−1(Rb)exp⁡(g−1(ln⁡(Vb)))π]1/2.

Therefore, the measurement of the size and shape of the bubble can be used to obtain information on the geometry of the nanowire and to reconstruct the size and shape of the nanowire.

## 5. Conclusion

The paper focuses on the forming and the evolution of the shape and size of the bubble through a photothermal process between a nanowire of TiO_2_ immersed in water and an electromagnetic wave. The increase of temperature is related to the geometry of the nanowire which leads to an increase in the shape and size of the bubble. That solution is computed by developing an adaptive remeshing method. That allows to compute with accuracy the temperature by adapting the mesh to the evolution of the bubble. The coupled problem (light, matter, heat) is solved through an adaptive loop process allowing converging to a stable solution and decreasing the number of nodes. The influence of the laser source and the geometrical parameters (wavelength, size, and shape of the nanowire) related to the size and shape of the bubble are presented and analyzed. The aspect ratio and the volume of the bubble can be expressed as function of the aspect ratio and the volume of the nanowire. By solving the inverse problem, two functions are obtained enabling finding the size and shape of the nanowire from the size and shape of bubble.

## Figures and Tables

**Table 1:** Fit parameters of the *f* function.

*λ*	NDP	NDF	FNI	Set of parameters			*σ* ^2^
				*a*_0_	*a*_1_	*a*_2_	
950	42	39	32	1.0036	0.2418	2.2334	1.8111*e* − 5
1000	38	35	15	1.0082	0.3659	2.6394	5.5850*e* − 5
1050	31	28	23	1.0124	0.6529	3.2416	1.1106*e* − 4
